# Advances in Traditional Chinese Medicine research in diabetic kidney disease treatment

**DOI:** 10.1080/13880209.2024.2314705

**Published:** 2024-02-15

**Authors:** Shiyi Shen, Huiyun Zhong, Xiaoshi Zhou, Guolin Li, Changji Zhang, Yulian Zhu, Yong Yang

**Affiliations:** a Department of Pharmacy, Sichuan Academy of Medical Sciences & Sichuan Provincial People’s Hospital, School of Medicine, University of Electronic Science and Technology of China, China; b School of Medicine and Food, Sichuan Vocational College of Health and Rehabilitation, Zigong, China; c School of Basic Medicine and Clinical Pharmacy, China Pharmaceutical University, Nanjing, China; d Department of Pharmacy, Ziyang People’s Hospital, Ziyang, China

**Keywords:** Chronic kidney disease, diabetic nephropathy, oxidative stress, clinical research

## Abstract

**Context:**

Diabetic kidney disease (DKD) is a prominent complication arising from diabetic microangiopathy, and its prevalence and renal impact have placed it as the primary cause of end-stage renal disease. Traditional Chinese Medicine (TCM) has the distinct advantage of multifaceted and multilevel therapeutic attributes that show efficacy in improving clinical symptoms, reducing proteinuria, protecting renal function, and slowing DKD progression. Over recent decades, extensive research has explored the mechanisms of TCM for preventing and managing DKD, with substantial studies that endorse the therapeutic benefits of TCM compounds and single agents in the medical intervention of DKD.

**Objective:**

This review lays the foundation for future evidence-based research efforts and provide a reference point for DKD investigation.

**Methods:**

The relevant literature published in Chinese and English up to 30 June 2023, was sourced from PubMed, Cochrane Library, VIP Database for Chinese Technical Periodicals (VIP), Wanfang Data, CNKI, and China Biology Medicine disc (CBM). The process involved examining and summarizing research on TCM laboratory tests and clinical randomized controlled trials for DKD treatment.

**Results and conclusions:**

The TCM intervention has shown the potential to inhibit the expression of inflammatory cytokines and various growth factors, lower blood glucose levels, and significantly affect insulin resistance, lipid metabolism, and improved renal function. Furthermore, the efficacy of TCM can be optimized by tailoring personalized treatment regimens based on the unique profiles of individual patients. We anticipate further rigorous and comprehensive clinical and foundational investigations into the mechanisms underlying the role of TCM in treating DKD.

## Introduction

**Figure 1.**
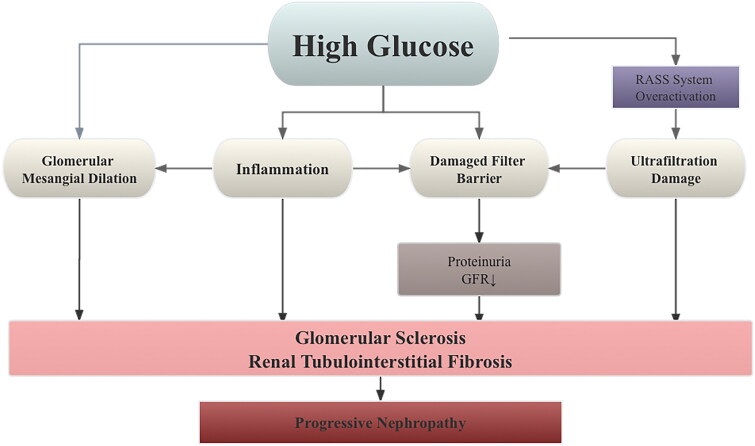
DKD is primarily caused by a number of interconnected mechanisms, including excessive filtration damage to the GFB, dilatation, and oxidation of the glomerular mesangium. These mechanisms are complex in nature, with links to hemodynamics, metabolism, and immunopathology. The physiological mechanism of DKD is summarized in this diagram.

Diabetic kidney disease (DKD) is a prevalent and severe complication of type 2 diabetes mellitus (T2DM), leading to various abnormalities in kidney structure, function, and clinical parameters (Marathe et al. 2017; Faselis et al. 2020). The onset of DKD is insidious, its progress is rapid, and the mechanism is complex. Reactive oxygen species (ROS) are the primary cause of vascular injury in diabetes mellitus, contributing to its development (Darenskaya et al. 2021). ROS is created excessively in hyperglycemia through a variety of mechanisms derived from both mitochondrial and cytoplasmic sources. As a result, increasing kidney cells’ antioxidant capability can help treat DKD. For instance, the nuclear factor erythroid 2-associated factor 2 (Nrf2), a fundamental regulator of cellular response to oxidative stress, is a viable therapeutic target. In addition, hyperglycemia can directly change proteins and cause glomerular filtration barrier permeability, mesangial dilatation, and glomerular inflammation in the context of oxidative stress and dyslipidemia. As a result, DKD patients’ nutritional status and dietary habits are important elements in treatment (Li et al. 2019; Hu et al. 2023). At the same time, increased susceptibility to oxidative stress due to increased glycolysis promotes cytokine-mediated mesangial dilation and aggravates ultrafiltration damage. In addition, abnormal activation of the renin-angiotensin-aldosterone system (RAAS) leads to constriction of efferent arterioles, resulting in increased glomerular pressure. The resulting hyperfiltration injury of glomerular fibrin B leads to decreased proteinuria and glomerular filtration rate (GFR). Therefore, the pathogenesis of DKD involving oxidative stress, metabolic abnormalities, and altered renal hemodynamics due to hyperglycemia (Toth-Manikowski and Atta 2015; Alicic et al. 2017; Tuttle et al. 2022), as summarized in Figure 1. Pathological changes such as proliferation of glomerular mesangial cells (MCs), thickening of the glomerular basement membrane (GBM), and buildup of extracellular matrix are the significant contributors to tubulointerstitial fibrosis (TIF), a critical phase in DKD development and other chronic kidney diseases leading to end-stage renal disease (ESRD) (Papadopoulou-Marketou et al. 2018; Huang F et al. 2019; Wang et al. 2021). Progression of DKD can result in persistent proteinuria and a decreased GFR, eventually leading to ESRD (Ritz and Orth 1999; Watanabe et al. 2016; Selby and Taal 2020). Without nephrology care, approximately 20% of patients with diabetic renal failure will require dialysis, imposing physical and financial burdens (Gao et al. 2023).

The prevalence of diabetes worldwide is rising, especially with the aging population, and approximately 40% of T2DM patients experience diabetes-related nephropathy (Stephens et al. 2020). It is estimated that by 2030, 643 million people worldwide will have diabetes. Approximately 1 in 10 Americans has diabetes, and 1 in 3 American adults has prediabetes. Up to 35% of people with diabetes will develop kidney disease (Lin and Erickson 2020). DKD has become the leading cause of chronic kidney disease (CKD) in China, surpassing glomerulonephritis. Over the years, China has witnessed a significant increase in diabetes prevalence, from 0.67% in 1980 to 12.8% in 2020, representing a 19-fold increase. Among these cases, combined DKD accounts for 20% to 40% (Cheng et al. 2023).

The American Diabetes Association (ADA) guidelines recommend using angiotensin-converting enzyme inhibitors/angiotensin receptor blockers in combination with glucose-controlling drugs as a therapeutic regimen to delay CKD progression by controlling proteinuria in patients with DKD (Samson et al. 2023). However, evidence-based medicine research suggests this approach does not significantly reduce the incidence of vascular events and mortality associated with DKD (Duni et al. 2019). Furthermore, it may not adequately meet the needs of patients with worsening renal function but normal leukocytosis or those with deteriorating renal function but normal leukocytosis (Afkarian et al. 2016; Krolewski et al. 2017). Despite stable glycemic control in diabetes patients, the incidence of CKD has not decreased in the past 20 years (Sulaiman 2019), necessitating the exploration of alternative and effective treatment options.

Traditional Chinese Medicine (TCM) alternative therapy offers several advantages: affordability, efficacy, fewer adverse effects, and better patient adherence. In recent years, research on TCM treatment for DKD has rapidly progressed. Previous research mainly studied or summarized one aspect of the clinical or experimental studies of TCM treatment of DKD, or did not distinguish the preclinical studies of TCM monotherapy and compound therapy from clinical studies. This review starts with the experimental study and clinical study of TCM monotherapy and compound therapy respectively, and summarizes the latest clinical and experimental progress of TCM treatment of DKD, aiming to provide basis and reference point for clinical prevention and treatment of DKD.

## Understanding DKD from the TCM perspectives

Traditional Chinese medical science provides a unique perspective on DKD, considering it a mixed syndrome of deficiency and excess (Wen et al. 2017). This deficiency dynamically changes as the disease progresses, with three distinct stages: the early stage, characterized by *yin* insufficiency and dry heat; the middle stage, with *qi* and *yin* insufficiency; and the later stage involving *yang* damaging by *yin*, eventually leading to *yang* deficiency or *yin* and *yang* deficiency. Superficial deficiency syndrome often presents symptoms such as dampness, blood stasis, and phlegm deficiency (Lu et al. 2019). DKD affects the kidneys but also causes disorders in the lungs, liver, spleen, and other organs.

In 2011, the Diabetes Branch of the Chinese Society of TCM established the Chinese Medicine Diagnosis and Treatment Standards for DKD, identifying three concurrent syndromes: *yin* deficiency and *yang* hyperactivity, blood stasis, and damp bladder heat (Gao et al. 2011). Blood stasis means that the blood flow is not smooth, the operation is blocked, and the stasis is in the meridians or organs. It plays a significant role throughout the development of DKD based on an analysis of 159 DKD articles, indicating its importance in the etiological mechanism of DKD (Sun C et al. 2015).

In contrast to Western medicine treatment, TCM therapy for DKD is characterized by multitarget approaches (Tang et al. 2021). Various combinations of Chinese herbal medicines can produce therapeutic effects from different points of view. Researchers have conducted clinical and experimental studies focusing on TCM therapy for DKD based on its etiological characteristics.

## Clinical investigation of TCM for DKD treatment

### Clinical research on the treatment of DKD with prescription formulas

Clinical studies have demonstrated the significant efficacy of TCM in treating DKD, as shown in Table 1. Many of these prescription formulas have been developed based on extensive clinical experience and have shown remarkable results in DKD treatment. Several studies have used the randomized controlled trial (RCT) method for validation.

**Table 1.** Clinical studies on the efficacy of prescription formulas in the treatment of DKD.

TCM	Composition	Number of enrolled patients	Intervention	Primary outcome	Study period (month)	Outcome	Reference
Tangshen Formula (TSF)	*Astragalus membranaceus* (Fisch.) Bunge*, Rehmannia glutinosa* (Gaetn.) DC.*, Panax notoginseng* (Burk.) F.H.Chen, *Cornus officinalis* Sieb. et Zucc.*, Euonymus alatus* (Thunb.) Sieb, *Rheum palmatum* L., *Citrus aurantium* L.	180	TSF vs. placebo	Change of urinary protein level	6	*P* = 0.024	(Yang et al. 2016)
Shenqi Dihuang decoction	*Cornus officinalis* Sieb. et Zucc.*, Codonopsis pilosula* (Franch.) Nannf., *Astragalus membranaceus* (Fisch.) Bunge*, Rehmannia glutinosa* (Gaetn.) DC*., Dioscorea opposita* Thunb.*, Alisma plantago-aquatica* Linn*., Poria cocos* (Schw.) Wolf*, Paeonia suffruticosa* Andr.	205	Shenqi Dihuang decoction vs. Metformin	m-ALB	3	*P* < 0.05	(Wang et al. 2018)
Buyang Huanwu decoction	*Astragalus membranaceus* (Fisch.) Bunge*, Angelica sinensis* (Oliv.) Diels.*, Radix Paeoniae* Rubra*, Ligusticum chuanxiong* Hort*., Carthamus tinctorius* L*., Prunus persica* (L.) Batsch	100	Buyang Huanwu Decoction vs. placebo	Change of UACR	6	*P* < 0.05	(Pan et al. 2022)
Zicuiyin decoction	*Astragalus membranaceus* (Fisch.) Bunge*, Rehmannia glutinosa* (Gaetn.) DC.*, Dioscoreaoppositifolia* L.*, Cornus officinalis* Siebold et Zucc.	88	Zicuiyin vs. placebo	Change of eGFR	2	*P* < 0.05	(Liu et al. 2022)

For example, an RCT confirmed the effectiveness of a Chinese herbal formula, the Bushen Jianpi Huoxue Recipe [consisting of *Angelica sinensis* (Oliv.) Diels 15 g, *Cistanche deserticola* Ma 10 g, *Cornus officinalis* Sieb. et Zucc. 12 g, *Cinnamomum cassia* Presl 8 g, *Paeonia suffruticosa* Andr. 12 g, *Alisma plantago-aquatica* Linn. 15 g], in treating DKD (Zhang et al. 2022), which exhibited positive effects on various clinical parameters, including glycosylated hemoglobin (HbAlc), fasting plasma glucose (FPG), blood pressure (BP), blood creatinine (SCr), urine albumin/creatinine ratio (UA/C), blood urea nitrogen (BUN), 2 h postprandial glucose (2hPG), and albumin/creatinine ratio (UACR), effectively addressing DKD (Higgins 2001; Nitin 2010; Idowu et al. 2017; Schnell et al. 2017; Xie Y et al. 2018; Colombo et al. 2020).

Innovatively, Li’s team proposed a ‘treating DKD from the liver theory’ and formulated the ‘Tangshen Formula,’ aimed at tonifying *qi*, softening the liver, promoting blood circulation, and unblocking collaterals (Zhao et al. 2016). This compound is getting a lot of attention. The Tangshen Formula demonstrated a significant reduction in urinary protein excretion and increased GFR, exceeding the effects of the placebo alone (Yang et al. 2016). At the same time, a 13-center RCT trial, registered NCT03009864, is being conducted in 632 participants to evaluate Tangshen Fang in patients with DKD by comparing changes in urine microalbumin–creatinine ratio from baseline to week 24 (Jin et al. 2019). In a multicenter RCT, 323 patients with stage III and IV DKD exhibiting *qi* and *yin* deficiency, along with crux and stasis syndromes, were randomly assigned to an experimental or control group. On the basis of the administration of basic Western medicine, patients in the experimental group were orally administered Yiqi Yangyin Xiaozheng Tongluo Formula, 1 dose per day, standard dosage of 150 mg/person, plus 150 mg of a placebo made of starch and saline, once a day. The control group was given 150 mg of oral irbesartan tablets daily and 150 mg of placebo, consisting of dextrin plus pigment and bitters. Placebo was given to both groups to simulate the different taste of Yiqi Yangyin Xiaozheng Tongluo Formula and irbesartan tablets in order to achieve the effect of double-blind experiment and obtain more objective experimental conclusions. After 24-month treatment, the results revealed that, compared to the positive control drug irbesartan, these formulas demonstrated significant improvements in the leading laboratory indicators of patients with stage III and IV DKD. Furthermore, this combined TCM treatment approach improved the patient’s living standards (Guo et al. 2021).

A meta-analysis involving 11 RCTs with 879 patients revealed that the experimental group, treated with Angelica Blood Replenishing Soup (ABRS) in addition to conventional treatment, exhibited a higher total effective rate, lower 24 h urine protein quantification, urinary albumin excretion rate, SCr, and BUN compared to the control group, concluding that ABRS was clinically effective as an adjuvant treatment for DKD (Cheng et al. 2021). Additionally, a single-center RCT study including 150 patients proved that on the basis of restricting dietary protein intake, combined with oral Buyang Huanwu Decoction 1 dose per day for 3 months, can also significantly improve the renal function indicators of patients, help regulate the level of inflammatory factors, and effectively improve the clinical efficacy of DKD (Chen et al. 2021).

### Clinical study of active ingredients in Chinese medicines for the treatment of DKD

In recent years, significant research has been conducted on the mechanisms of active ingredients in TCM to treat DKD. A meta-analysis of RCTs involving 829 patients found that combining Tripterygium glycosides with valsartan had a limited impact on SCr levels but showed an improved reduction in 24 h urine protein levels in DKD patients (Ye et al. 2019). Another meta-analysis of 35 RCTs comprising 2,320 patients with DKD examined the effects of breviscapine injection. The findings demonstrated that breviscapine had a nephroprotective and lipid-modulating effect in DKD patients, reducing urinary protein, SCr, cholesterol, and triglyceride levels compared to control groups (Liu et al. 2016).

**Figure 2.**
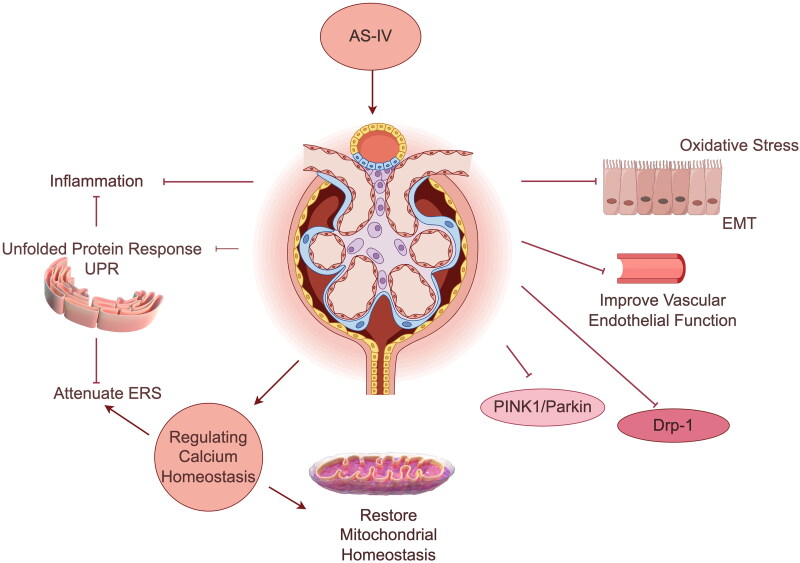
AS-IV may treat DKD by reducing inflammation, attenuating ERS, regulating calcium homeostasis, restoring mitochondrial homeostasis (*via* the Drp-1 and PINK1/Parkin signaling pathways), countering oxidative stress, and improving EMT and vascular endothelial function.

Recent studies have provided evidence supporting the efficacy of *Astragalus membranaceus* (Fisch.) Bunge in managing DKD. *Astragalus* has been shown to effectively reduce urinary protein, improve renal function, and control the progression of DKD (Liu et al. 2020). Its active ingredients, including total *Astragalus* flavonoids, astragaloside A, and *Astragalus* polysaccharide, have been found to meliorate DKD by combating oxidative stress, inhibiting dynamin-related protein 1 (Drp-1) and PTEN-induced putative kinase protein 1/E3 ubiquitin-protein ligase parkin (PINK1/Parkin) signaling pathways to restore mitochondrial homeostasis, weakening endoplasmic reticulum stress (ERS), regulating calcium homeostasis, reducing inflammation, and improving epithelial-mesenchymal transition (EMT) and vascular endothelial function (Lu et al. 2013; Cui et al. 2016; Li et al. 2018; Wang et al. 2018; Wang et al. 2020; Gao et al. 2023). The relevant mechanism is shown in Figure 2. In addition to *Astragalus*, there are numerous other active herbal ingredients with demonstrated clinical efficacy in managing DKD (Wang et al. 2012, 2015; Zhu et al. 2016; Voroneanu et al. 2017; Sattarinezhad et al. 2019; Wang et al. 2019; Chang et al. 2020).

### Clinical study of Chinese patent medications for DKD therapy

Numerous studies have provided strong evidence for the efficacy of Chinese patent medications in managing DKD (Li et al. 2015; Lin L et al. 2016; Liu et al. 2016; Xiang et al. 2016; Zhao et al. 2019). One clinical trial used a multicenter randomized, double-blind, placebo-controlled design and involved 120 patients with DKD. The experimental group received *Cistanche deserticola* kidney capsules, while the control group received a placebo for six months. The results showed that the experimental group indicated better outcomes in alleviating clinical symptoms and reducing proteinuria, confirming the efficacy of *Cistanche deserticola* kidney capsules (Fang et al. 2012). In a retrospective investigation, the total clinical effective rate of the irbesartan-treated control group was only 74.5% (Dai et al. 2023). However, in the experimental group of DKD patients treated with Jinlida granules combined with irbesartan, the total clinical effectiveness rate was significantly higher at 90.9%. Furthermore, the experimental group showed lower clinical symptoms scores for head and body drowsiness, facial swelling, fatigue, and epigastric distension, indicating that Jinlida granules combined with irbesartan had a more substantial positive impact on clinical symptoms in DKD patients. Moreover, a meta-analysis of 26 RCTs involving 4,676 patients (2,342 in the experimental group and 2,334 in the control group) evaluated the therapeutic efficacy of Jinshuibao capsules (JSBC) in the early intervention of DKD. The analysis revealed that JSBC significantly reduced the urinary microprotein excretion rate (UAER) in DKD patients and improved indexes such as urinary protein, HbA1C, SCr, and FPG, indicating its effectiveness in early DKD intervention (Yu et al. 2022).

In conclusion, numerous clinical investigations have demonstrated the potential of TCM in treating DKD. However, these studies have some limitations that warrant consideration. First, all of the clinical research included in this article is conducted in China, primarily in hospital settings, and more than half of the included studies had no detailed information on randomization methods, allocation concealment, and blinding techniques. Moreover, the cited meta-analysis had multiple outcome measures but only reported statistically significant results, which may lead to reporting bias. So, the impact of potential selection bias is unclear. This high risk of bias and selective bias could affect the reliability of the meta-analyses-type study results. At the same time, it also brings difficulty to the promotion of the research, and further verification is needed in other countries and regions. Second, many trials have small sample sizes and include a diverse population, making it challenging to generalize the results universally. Third, the lack of reporting on adverse effects in most RCTs hinders the evaluation of treatment safety. Furthermore, the short duration of follow-up (1–3 months) and the absence of endpoints, such as the incidence of ESRD, morbidity, mortality rates, and survival quality, limit the assessment of long-term efficacy. To improve understanding of TCM treatment for DKD, future RCTs should adopt larger sample sizes, rigorous trial designs, longer follow-up periods, and more comparable studies. Comprehensive investigations of adverse reactions and other relevant indicators in DKD treated with TCM are also necessary to address safety concerns.

### Experimental study on the treatment of DKD by TCM

DKD imposes a significant economic burden on society. Pang et al. (2019) conducted studies to explore the therapeutic mechanisms of TCM in DKD. Their research highlighted the anti-inflammatory, antioxidant, lipid regulation, and anti-glucose effects of TCM in managing DKD. The disease progression of DKD is influenced by three main factors: excessive filtration damage to the glomerular filtration barrier (GFB), dilation of glomerular tethered membranes, and oxidative stress, which are associated with hemodynamic, metabolic, and immunopathological factors (Schena and Gesualdo 2005; Barrera-Chimal and Jaisser 2020; Bonner et al. 2020; Tuttle et al. 2022). Despite considerable research, the precise pathophysiology of DKD still needs to be understood. Conventional therapeutic drugs, such as hypoglycemic, antihypertensive, and lipid-lowering drugs, have limitations in effectively delaying or controlling the development of DKD (Zhong et al. 2019).

Over the past several decades, TCM has demonstrated promising therapeutic effects for treating DKD in clinical settings. However, there needs to be more in-depth research on the underlying mechanisms through animal model experiments. In recent years, TCM practitioners have conducted a series of experimental studies, integrating their understanding of the etiology and pathogenesis of DKD with modern medical concepts. Investigating the mechanism of TCM in DKD treatment offers several benefits. It allows for customized and individualized treatment plans, considering each patient’s unique pathogenesis. This targeted approach can lead to more effective and timely control of disease progression, optimizing patient outcomes.

## Experimental study on the treatment of DKD with single TCM

### Modulation of endoplasmic reticulum stress

Oxidative stress promotes the development of DN. Reactive oxygen species (ROS) play a crucial role in mediating oxidative stress and ERS (Jha et al. 2016). In recent years, the regulatory role of ERS in the pathogenesis of DKD has received considerable attention. Guo et al. (2016, 2017) conducted experiments using spontaneous and induced diabetes animal models, such as db/db mice and streptozotocin-induced diabetes mice. They observed that protein kinase RNA-like ER kinase (PERK) (Bao et al. 2021), inositol that requires enzyme 1α (IRE1α) (Xie et al. 2022), and activating transcription factor 6 (ATF6) (Tang et al. 2011), along with their downstream targets, were activated, leading to a significant increase in the expression of the ERS marker CCAAT/enhancer binding protein homologous protein (CHOP) and factors such as c-Jun N-terminal kinase (JNK) (Yung and Giacca 2020) and caspase-12 (Brezniceanu et al. 2010), which induce cell apoptosis. Interestingly, the chemical component Astragaloside IV (AS-IV) found in *Astragalus membranaceus* has shown the ability to reverse this situation. AS-IV reduces ERS and cell apoptosis in DKD animal models. Astragaloside significantly improved urinary albumin excretion and SCr and BUN levels in DKD rats while inhibiting podocyte apoptosis (Wang et al. 2015). The effect was independent of the hypoglycemic effect and was related to the downregulation of PERK, which reduced ERS.

Puerarin can also delay the progression of DKD by resisting ERS (Bai et al. 2021). Puerarin has been identified to have hypoglycemic effects. Its mechanism of action may involve inactivation of Janus kinase 2 (JAK2)/signal transducer and activator of transcription 3 (STAT3) signaling in pancreatic tissues of DKD rats, inhibiting the PERK-Eukaryotic initiation factor 2 alpha (eIF2α)-activating transcription factor 4 (ATF4)-CHOP pathway and mitigating apoptosis of islet β-cells (Hu et al. 2021). Furthermore, puerarin exhibits a protective effect on renal podocytes in streptozotocin-induced DKD rats, leading to reduced levels of autophagy markers such as PERK, ATF4, eukaryotic translation initiation factor 2α kinase (EIF2α), and Beclin-1 in renal tissues. This, in turn, increases the expression of functional podocyte proteins. The observed outcomes may be related to the activation of the PERK/eIF2α/ATF4 signaling pathway, regulating the ERS-related autophagy pathway (Xu et al. 2020).

### Inhibition of cell death

In DKD, a critical aspect of pathological alterations involves damage to tethered cells, unique smooth muscle cells located throughout the capillary loops of the glomerular capillaries (Watanabe et al. 2016). Various modes of cell death, including apoptosis, autophagy, ferroptosis, necrosis, and pyroptosis, have been identified in different physiological and pathological contexts (Bertheloot et al. 2021). Ferroptosis is a recently recognized form of programmed cell death characterized by an unexpected increase in oxygen lipid free radicals within cells (Dixon et al. 2012; Jiang et al. 2021; Shen et al. 2022). Some active Chinese herbal ingredients have shown the potential to treat DKD by targeting ferroptosis suppression, thus attenuating high glucose and diabetes-induced tubular and glomerular injury and renal fibrosis. Understanding and targeting these processes, such as ferroptosis and tethered cell damage, can offer novel therapeutic approaches for the treatment of DKD, highlighting the potential of Chinese herbal medicine to address the complex pathogenesis of this condition.

*In vitro* experiments have revealed that high glucose and keratin significantly induce the release of lactate dehydrogenase (LDH) and the expression of long-chain acyl-coenzyme A synthetase 4 (ACSL4), NADPH oxidase 1 (NADPH 1), and cyclooxygenase 2 (COX2). Furthermore, they also reduce glutathione peroxidase 4 (GPX4). Interestingly, using an iron chelating agent can reverse glucose-induced changes, including LDH release and the expression of ferroptosis-related genes in mice mesangium cells. These findings indicate that high glucose can induce ferroptosis in mesangial cells through podocyte injury (Yang et al. 2021).

Ferroptosis has been associated with tubular cell death in DKD (Wang et al. 2020). Calycosin (CAS), a tricholoma isoflavone derived from the rhizome of *Astragalus membranaceus*, has demonstrated properties such as immunomodulation (Zhang et al. 2012), anti-inflammatory effects (Hoo et al. 2010), antiviral activity, and antioxidant capabilities (Nie et al. 2016). *In vitro* experiments investigated the impacts of CAS. They found that CAS mitigated the decline in the viability of human renal tubular epithelial cells (HK-2 cells) under high glucose conditions. Besides, the dose-dependent effect of CAS on increasing cell viability was proportional. In an *in vivo* model experiment, CAS was administered to db/db mice at 10 or 20 mg/kg/d doses, while db/m mice were used as a control group. Histological examination using Hematoxylin and Eosin (HE) staining revealed that the experimental group of mice treated with CAS exhibited less renal tubular damage. In contrast, the control group showed severe renal injury. These results suggest that CAS effectively protects renal function and mitigates diabetes-induced renal injury in experimental mice (Huang et al. 2022).

Further research on the two key indicators of ferroptosis, GPX4, and nuclear receptor coactivator 4 (NCOA4), revealed that CAS could raise ROS and NCOA4 levels while promoting the increase in GPX4. However, keratin could block this effect, suggesting a possible connection between the mode of action of CAS and ferroptosis. These experimental findings shed light on the potential protective effects of the natural active ingredients found in TCM on the kidneys and their underlying mechanisms. This research opens up new possibilities for treating DKD using TCM-based approaches.

## Experimental study on the treatment of DKD using a compound formula

### Modulation of the inflammatory response

**Figure 3.**
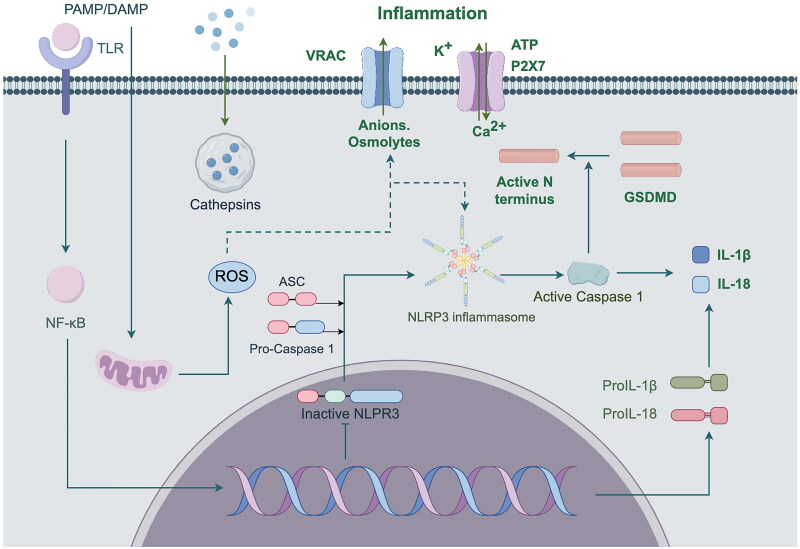
ROS, ATP and other substances trigger NF-κB dependent pro-IL-1β and pro-IL-18 gene transcription by activating TLR/IL-1 receptors. After Caspase-1 cleavage, these pro-inflammatory factors are released into the cytoplasm in the form of pro-IL-1β and pro-IL-18, thus participating in the inflammatory response.

Inflammatory response is the most common phenotype in all progression of DKD. In terms of inflammatory response, TCM mainly acts by regulating the NF-κB signaling pathway, TNF-α signaling pathway, IL signaling pathway, NLRP3 signaling pathway and MAPK signaling pathway (Hu QC et al. 2023). Inflammatory damage orchestrated by nucleotide oligomerization domain (NOD)-like receptor protein 3 (NLRP3) inflammasomes is intricately linked to the TCM concepts of *qi* and *yin* deficiency, blood stasis, and toxin (Huang et al. 2019). NLRP3 is an important universal sensing protein involved in immune response by regulating the maturation and secretion of pro-inflammatory cytokines (Shahzad et al. 2022). Long-term high glucose will promote the activation of NLRP3 inflammasome, activated NLRP3 inflammasome activates Caspase-1, Caspase-1 shear IL-1β and IL-18 precursors, and activate them, thereby regulating IL-18 and IL-1β. IL-1β and IL-18, both members of the interleukin-1 family, are important pro-inflammatory cytokines that can cause local and systemic inflammatory responses (Liu et al. 2021). The NLRP3 inflammasome pathway is shown in Figure 3. Therefore, Activation of NLRP3 inflammasomes within the body can trigger inflammatory responses, disrupt organ function, and cause various pathological alterations that exacerbate the progression of DKD. Zhu et al. (2022) demonstrated that different doses of Danggui buxue decoction and its main component Astragaloside can effectively inhibit the upregulation of NLRP3 and Caspase-1 in DKD model rats, and improve renal function and adipocyte injury in GK rats by blocking the activation of NLRP3 inflammasome, thus playing a renal protection role.

In db/db diabetic mice, various doses of the Wumei pill exhibited a dose-dependent reduction in FPG levels while enhancing serum insulin secretion (Yang et al. 2019). In particular, in the pancreatic islet tissues of model mice, the Wumei pill showed protective effects on pancreatic β-cells. This protection was demonstrated through attenuation of caspase-12 patterns, enhancement of Bcl-2 expression, reduction in the generation of IL-1β, IL-18, monocyte chemotactic protein-1α, and macrophage-specific surface glycoproteins F4/80. Furthermore, the Wumei pill modulated the protein expression levels of key components of the NLRP3 inflammasome of islet tissue, such as apoptosis-associated speck-like protein and caspase-1. Consequently, the authors propose that the Wumei pill hindered the activation of NLRP3 inflammasomes, effectively protecting pancreatic cells and impeding the progression of T2DM.

Previous research has indicated that Fufang Zhenzhu Tiaozhi capsules (FTZ) can modulate blood glucose levels, lipid metabolism, and inflammatory reactions while protecting pancreatic β-cells (Hu et al. 2014; Chen et al. 2018). Yang et al. (2022) conducted an experiment using a DKD mouse model, where the experimental group received FTZ at a dose of 1.2 g/kg/day, and the positive control group received the same dose of FTZ along with chlorosartan (30 mg/kg/day) for 12 weeks. The study revealed that the experimental group exhibited lower levels of 24 h urine protein, SCr, FPG, total cholesterol, triglycerides, and low-density lipoprotein cholesterol (LDL-C) compared to the model control group.

Emerging evidence from studies suggests that IL-17A contributes to renal inflammation by activating the nuclear factor κB (NF-κB) signaling pathway (Lavoz et al. 2019; Ma J et al. 2019; Warren et al. 2019). In this particular investigation, FTZ demonstrated the ability to significantly inhibit fibronectin growth and type IV collagen aggregation, and suppress interstitial mesangial cell expansion in the glomerular and tubular, the infiltration of F4/80^+^ macrophages, and Ly-6G^+^ neutrophils. Furthermore, FTZ inhibited the IL-17A and NF-κB signaling pathways. Consequently, FTZ could exert its effects by dampening IL-17A expression *in vivo*, potentially slowing the progression of DKD and decreasing renal inflammatory responses and fibrosis.

### Treatment of foot cell injuries

Earlier investigations (Dai et al. 2009; Kanwar et al. 2011; Zhou et al. 2019; Schunk et al. 2021; Mo et al. 2022) have illuminated that the Wnt4/β-catenin pathway can contribute to the pathogenesis of DKD by mediating podocyte injury and glomerulosclerosis. Combining tonification of *Yang* and restoration of five soups with ginseng and *Astragalus* Dihuang soup is believed to improve *qi* and nourish *yin*, facilitate blood circulation, alleviate symptoms, and clear collaterals. Previous studies (Matsui et al. 2007; Dai et al. 2009) have indicated that this combination can raise E-cadherin levels and down-regulate the expression of proteins such as Wnt4, glycogen synthase kinase 3β (GSK3β), β-catenin, transforming growth factor-β1 (TGF-β1), type III collagen (Col-III), fibronectin, and smooth muscle actin (α-SMA). These findings suggest its potential to inhibit the Wnt4/β-catenin pathway, leading to an improved EMT of renal tubular epithelial cells (TECs) and subsequently reducing tubulointerstitial fibrosis (TIF). In a study, Tang Shen Ning positively affected *qi* nourishment, *yin* enhancement, stasis elimination, and stagnation dissipation (Cui et al. 2021). This formula was found to up-regulate P-cadherin, synaptopodin, and nephrin levels, while down-regulating the expression of β-catenin, Desmin, Col-I, Snail, and fibroblast-specific protein 1 (FSP-1). These effects helped prevent the EMT of podocytes, thus mitigating TIF and safeguarding podocytes from DKD-induced damage. Furthermore, TCM granules have been reported (Fang et al. 2016; Lu et al. 2017) to inhibit Wnt4/β-catenin pathway activation by improving the protein expression levels of podocyte cleavage membrane proteins (Podocin, Nephrin, CD2AP). This inhibition was associated with better podocyte health, reduced proteinuria, delayed glomerulosclerosis, and fibrosis.

In a retrospective cohort study (Chan et al. 2022), it was observed that R-6 contributed to delay changes in pathological renal function in DKD patients and also led to a decrease in mortality rates. Based on this investigation, Chan et al. (2022) employed a system pharmacology approach to investigate the results. This analysis revealed that among all variants of R-6, a significant cluster was centered on tumor necrosis factor (TNF) as its core component. The conclusion was that the primary impact of R-6 on DKD operates through the TNF signaling pathway, which serves as a central mechanism to mitigate podocyte damage. Furthermore, R-6 exhibited the ability to maintain the stability of the expression of nephrin and podocin in podocytes from DKD rats, preserving the structural and functional integrity of these cells. As a result, it actively contributed to reducing urinary protein and proactively worked to decelerate the progression of DKD.

### Antioxidant stress

Oxidative stress plays a significant role in the progression of DKD (Jha et al. 2016). Recently, a treatment approach based on the core concept of antioxidant stress in TCM has been implemented in clinical practice for DKD (Roumeliotis et al. 2021). Research has indicated that TCM interventions effectively reduce oxidative stress levels associated with DKD and exert a therapeutic influence by regulating various oxidative stress signaling pathways (Sun G-d et al. 2016).

In conditions of chronic hyperglycemia, the kidneys activate various pathways, including the advanced glycosylation end product (AGE)/AGE receptor (RAGE) pathway (Kawarada et al. 2016), Nrf2 pathway (Ma et al. 2021), protein kinase C pathway, hexosamine pathway, and NAPDH oxidase pathway. These pathways ultimately enhance oxidative stress, damaging renal tissues, and deteriorating renal structure and function. This damage includes glomerular hypertrophy, dilatation of the glomerular plasma membrane, basement membrane thickening, endothelial dysfunction, and extracellular matrix deposition (Ighodaro 2018; Volpe et al. 2018).

The Kelch-like ECH-associated protein 1 (Keap1)-Nrf2-antioxidant responsive element (ARE) signaling pathway is recognized as a pivotal mechanism within the body’s antioxidant defenses (Hernandez et al. 2022; Tanase et al. 2022; Robertson 2023). Nrf2 is a transcription factor that plays a role in countering oxidative stress by inducing gene expression associated with antioxidant processes (Zheng et al. 2011; Uruno et al. 2015; Cheng et al. 2019). The compound Centella (a blend of *Centella asiatica* (L.) Urb., *Astragalus membranaceus*, and *Rehmannia glutinosa* (Gaetn.) DC.) could reduce the protein/creatinine ratio in DKD rats by upregulating the expression of Keap1 and Nrf2-related genes and proteins in the kidneys, thus slowing the progression of DKD (Zhu et al. 2020). Heme oxygenase-1 (HO-1), a critical anti-inflammatory enzyme that protects against oxidative and harmful chemicals (Sousa et al. 2017; Zhang et al. 2019), can be enhanced by the compound centella, suggesting a possible link between the protective effect of centella in DKD rats and the Keap1-Nrf2-ARE pathway under oxidative stress. AGEs are stable metabolites produced by macromolecules under non-enzymatic conditions during prolonged hyperglycemia.

Danggui-Shaoyao-San (DSS) improved AGE levels and their associated renal lipid peroxidation products in DKD rats (Liu IM et al. 2012). These findings suggest that DSS not only decreases AGE expression in diabetic glomeruli by managing blood glucose levels but also potentially acts through antioxidant activity. TCM might slow the progression of DKD by modulating the equilibrium of various oxidative stress markers.

## Conclusions

The pathogenesis of DKD remains complex, involving various factors such as altered glucose metabolism, hemodynamic irregularities, lipid metabolism imbalances, inflammation, oxidative stress, and more, all interconnected and influencing each other. As a result, it is crucial to consider the comprehensive treatment process rather than focus solely on isolated indices. TCM compounds, individual TCM herbs, and active ingredients possess the advantage of exerting multi-target effects. The therapeutic mechanisms for DKD encompass the modulation of podocyte-related proteins, signaling pathways, anti-inflammatory actions, and antioxidative stress effects. These mechanisms serve as foundational references for TCM diagnosis and treatment. However, larger-scale multicenter studies are lacking, and discrepancies in therapeutic mechanisms among different TCM syndromes must be clarified. Thus, future research demands more clinical trials and experimental studies. It is expected that as research on DKD treatment with TCM deepens, additional scientific evidence will emerge, significantly improving the guidance for clinical treatment and prevention. Simultaneously, this progress will strengthen the theoretical basis for the prevention and therapy of DKD, ultimately advancing the standardization and normalization of TCM approaches for DKD prevention and treatment.

## Data Availability

This review uses data from a variety of sources, including PubMed, Cochrane Library, VIP, Wanfang Data, CNKI, and CBM, etc.
